# Is Black Always the Opposite of White? An Investigation on the Comprehension of Antonyms in People with Schizophrenia and in Healthy Participants

**DOI:** 10.3390/bs5010093

**Published:** 2015-03-09

**Authors:** Cristina Cacciari, Francesca Pesciarelli, Tania Gamberoni, Fabio Ferlazzo, Leo Lo Russo, Francesca Pedrazzi, Ermanno Melati

**Affiliations:** 1 Department of Biomedical, Metabolic and Neurological Sciences, University of Modena, Via Campi 287, Modena 41100, Italy; E-Mail: cristina.cacciari@unimore.it; 2 Centro Salute Mentale, Via Martiri 63, Pavullo 41126, Italy; E-Mail: t.gamberoni@ausl.mo.it; 3 Villa Igea Private Hospital, Via Stradella 73, Modena 41100, Italy; E-Mail: leolorusso@villaigea.it; 4 Department of Psychology, University of Rome, Via dei Marsi 78, Rome 00185, Italy; E-Mail: fabio.ferlazzo@uniroma1.it; 5 Centro Salute Mentale Polo Ovest, Via Newton 150, Modena 41126, Italy; E-Mails: f.pedrazzi@ausl.mo.it (F.P.); e.melati@ausl.mo.it (E.M.)

**Keywords:** paranoid schizophrenia, language comprehension, antonym, semantic relationship

## Abstract

The present investigation sought to expand our understanding of the cognitive processes underlying the recognition of antonyms and to evaluate whether these processes differed in patients with schizophrenia and in healthy controls. Antonymy is the most robust of the lexico-semantic relations and is relevant to both the mental organization of the lexicon and the organization of coherent discourse, as attested by the resurgence of interest in antonymy in the linguistic and psychological domains. In contrast, the vast literature on semantic processing in schizophrenia almost ignored antonymy. In this study, we tested the online comprehension of antonyms in 39 Italian patients with paranoid schizophrenia and in an equal number of pairwise-matched healthy controls. Participants read a definitional sentence fragment (e.g., the opposite of black is), followed by the correct antonym (white) or by a semantically unrelated word (nice), and judged whether or not the target word was correct. Patients were rather accurate in identifying antonyms, but compared to controls, they showed longer response times and higher priming scores, suggesting an exaggerated contextual facilitation. Presumably, this reflects a deficient controlled semantic processing and an overreliance on stored semantic representations.

## 1. Introduction

Schizophrenia (SZ) is a neurobiological disorder associated with several affective, behavioral and cognitive deficits (DSM-V) that include mild to severe language comprehension and production abnormalities (for overviews, see [1,2,3,4]), as well as attentional and information processing impairments [5,6]. Brain-imagining studies revealed that SZ is associated with abnormality of a network of brain areas (e.g., a reversed laterality of activation in the superior temporal gyrus, morphological asymmetries in the superior temporal lobe, structural abnormalities of the ventral parts of the prefrontal cortex) that include the frontal and temporal cortex, the hippocampus and subcortical regions (for overviews, see [7,8,9]). The brain areas with abnormal activation and/or morphology partially overlap with the areas necessary for language comprehension. In fact, a wealth of studies on SZ have documented language comprehension deficit [1,2,10,11,12,13]. Language comprehension impairments are not global and generalized and can selectively involve abnormalities at a word and/or sentence level. At a word level, semantic investigations on people with SZ have mostly relied on the semantic priming paradigm (semantic priming occurs whenever the response to a target is facilitated by prior presentation of a related prime). These studies have obtained mixed results, showing increased, normal and decreased priming effects (for overviews, see [1,2,10,11,12,13]). Predominantly, a greater than normal semantic priming (hyperpriming) has been observed at short intervals between the presentations of prime and target (SOA, stimulus onset asynchrony) especially, but not only, in thought-disordered patients. Hyperpriming was often accompanied by reduced or absent priming at long SOAs (more than 300 ms). These abnormal priming effects would emerge from a distorted neural processing of the relationships between concepts in long-term semantic memory and from functional abnormalities of semantic memory neural networks (for overviews, see [10,11,14]) that would lead to abnormally fast and/or far-reaching spreading of activation among concepts [14,15]. In addition, patients would fail to suppress or deactivate these associations, even when they are contextually inappropriate [16]. In fact, another hallmark of SZ is a distorted use of context in linguistic and non-linguistic processing [4,17,18,19,20,21,22] that has been thought to reflect a more general inability of patients to construct and maintain an internal representation of context for the control of action [23], due to working memory deficit [4,17,24,25]. However, according to a different interpretation of contextual deficits, SZ patients would fail at inhibiting contextually-irrelevant information, especially for long SOAs [10], rather than in encoding contextually-relevant information due to a more global deficit in controlled semantic processing [21,22]. In sum, it seems that although SZ does not necessarily lead to a loss of lexico-semantic knowledge [26], it may nonetheless impair the ways in which this knowledge is stored, retrieved and used [1,2,15,26,27,28].

Deficits in processing sentential meaning have been observed at different levels that span from syntactic to pragmatic levels (for overviews, see [26,29]). For instance, it has been reported that SZ patients can be insensitive to semantic anomalies, presumably because of deficits in keeping active context during online processing [26], or impaired in establishing referential links because of the interference coming from the sustained activation of contextually inappropriate lexico-semantic associations [16].

### 1.1. Antonym Word Pairs

Conceptual knowledge stored in semantic memory encompasses representations of many different types of lexico-semantic relationship, among which is antonymy [30]. The term “antonymy” is generically used to refer to any of two words that are semantically opposed and incompatible for at least one of their senses [31] (e.g., black/white, dead/alive, long/short, optimistic/pessimistic). In this study, as in other studies [31,32], we are using “antonymy” as a cover term for all different kinds of opposites.

Antonyms occur very frequently in written and oral language, presumably because binary contrast is a powerful organizing principle in perception and cognition [32,33]. Antonymy is thought to be the most robust of the lexico-semantic relations, relevant to both the mental organization of the lexicon and the organization of coherent discourse [31,34,35,36,37]. In fact, it has been proposed that antonyms function as hubs around which the adjective vocabulary revolves [32].

Antonym word pairs lexicalize a fundamental type of semantic relationship. Therefore, they represent an important phenomenon for elucidating not only the nature of the semantic dysfunction that characterizes SZ, but on more general grounds, for establishing the neural and cognitive prerequisites of word storage and comprehension. Studying the types of semantic relationships that patients with SZ can or cannot correctly comprehend can also yield further insights into the organization of semantic knowledge in the human brain and into the mechanisms underlying its use [2]. In fact, the importance of antonyms for elucidating the organization and retrieval of semantic knowledge is documented by the recent resurgence of linguistic and psycholinguistic studies on this topic (for overviews, see [38,39]). This renewed interest concerns language-unimpaired comprehenders and stands in sharp contrast to the fact that the vast majority of neuropsychological studies on conceptual representations has primarily investigated semantic similarity rather than opposition [30], despite the fact that semantic opposition, rather than similarity, is thought to be the axis around which the adjectival lexicon clusters [32,36,39].

The vast literature on semantic processing abnormalities in SZ has almost ignored antonyms. The few paper-and-pencil studies of the 1960s [40,41] have documented that patients failed in distinguishing antonyms, synonymous and homonymous words in multiple choice tasks and selected antonyms or homonyms, rather than synonyms (or unrelated words), in response to a word stimulus. These errors were linked to semantic biases associated with illogical thinking and loose associations. Neuropsychological studies on pathologies different from SZ obtained a partly different picture. For instance, a recent study on global aphasics [30] reported a spared ability to identify antonyms, but not synonyms. This difference was interpreted by the authors as reflecting the fact that aphasic patients elaborated polarity information more efficiently than other aspects of word meaning, presumably because while the identification of fine-grained semantic differences in synonyms required an in-depth semantic processing. In contrast, “task demand may be lower when one has to identify one single large discrepancy than multiple small discrepancies. Such difference may have greater salience” [30] (p. 2642).

Deciding whether or not the opposite of black is white seems a rather demanding task for people with SZ [40,41], but it is instead a low demanding task for healthy participants [38,39,40,41,42,43,44]. In fact, antonyms are recognized faster than any other words or non-words in word recognition, elicit each other in word association tests (constituting approximately a quarter of the responses coded in free association norms [30,45]) and are often mistaken in speech error analyses (making up approximately 30% of word substitution errors of healthy participants [36,41,46]).

### 1.2. The Present Study

Given the relevance of this high order semantic relationship, shedding light on whether or not antonym identification is spared in a neurobiological disorder typically associated with semantic deficit may improve our understanding of the organization of word storage and retrieval in the human brain [47]. The general aim of this study was therefore to expand our understanding of the cognitive processes underlying the recognition of antonyms and to evaluate whether these processes differed in people with SZ and in healthy controls. We tested whether the semantic dysfunction that often characterizes people with SZ necessarily leads to a loss of the capacity to recognize antonyms when antonyms are presented alone, rather than with homonyms and/or synonyms, as in prior studies [39,40], and when antonym identification is tested with a real-time task.

In this study we used a target verification task modeled after the task used to test antonym comprehension in language-unimpaired participants [43,44]. Participants were presented with a definitional sentence fragment containing the first word of the antonym pair (e.g., the opposite of black is), followed by the correct antonym (white) or by a semantically unrelated word (nice), and had to decide whether or not the target was correct. This task is suited to obtain information on real-time comprehension, while at the same time placing little demand on the need to maintain and update information in working memory. The presentation of the definitional sentence fragment and of the target followed a self-paced pressing of the space bar, rather than being *a priori* decided at fixed rates. This choice is motivated by the fact that self-paced methods are known to allow subjects to read at a pace that matches their internal comprehension processes [48,49]. In addition, using similar, fixed time durations for patients and controls would have been problematic, since SZ patients typically need longer presentation durations to perceive a stimulus [50,51,52].

We expected healthy subjects to respond in fast and accurate ways, in line with the literature. If people with SZ are impaired in processing antonyms, what would the results look like? It is well-known that SZ patients tend to be less accurate and slower than healthy controls on most cognitive measures [5,53]. Since response slowing is related to the disease process, rather than necessarily reflecting semantic dysfunction [20], this may lead to an artificial increase of the reaction time difference with healthy participants that questions the interpretation of a group difference in terms of processing deficit. To avoid the confound represented by the slowed response pace of patients, semantic priming studies (e.g., [54,55,56]) have proposed to use a relative priming score (PRI; [54]) (see the Methods Section), rather than the mere response times to the targets. The priming score reflects the amount of facilitation of prior context on the response times to a target. Hence, we compared the individual priming score of patients and controls in response to antonyms and non-antonyms. Semantic priming studies often observed an exaggerated priming score of patients compared to controls (for an overview, see [2]) that has been mostly attributed to faster than normal and a far-reaching spread of activation in semantic memory. This heightened semantic priming effect has been observed under the “automatic” condition that typically characterizes word priming for short SOAs [10]. In this study, the priming effect elicited by the definitional sentence fragments on target words, if any, would occur under strategically controlled conditions, since the target presentation is self-paced, and the definitional sentence fragment strategically guides the semantic search toward the item that fulfilled the antonymy definition. This notwithstanding, if indeed people with SZ are characterized by an abnormal spread of activation, we may obtain heightened priming scores in patients, compared to controls. Finding a heightened priming effect also in strategic conditions would represent *per se* an interesting contribution to the study of semantic priming effects.

The easy nature of the task, the high written frequency and bound lexical couplings of the antonym pairs we used (see the high cloze reported in the Methods Section) can minimize semantic processing demands. However, it is unlikely that an even intact ability to identify antonyms would eliminate any group difference in performance in the face of the general cognitive deficits that typically characterize people with SZ. To circumvent this problem and limit the potential confound represented by variables associated with the cognitive state of the patients, we conducted analyses of covariance on mean response times and accuracy to partial out the contribution of a set of covariates (*i.e.*, verbal fluencies, vocabulary and digit span).

The general accuracy of patients may not be necessarily compromised, given the mild-to-moderate form of SZ, the low demanding nature of the task and the high familiarity and boundedness of the antonym pairs. However, accuracy can be modulated by the severity of thought disorder. In fact, prior studies [8,11,16,57,58,59] have documented that the abnormal semantic processing in SZ is often closely associated with positive thought disorders, a multidimensional disturbance that emerges in both language comprehension and production [4]. Hence, patients scoring higher in scales measuring thought disorders may also have a lower accuracy and/or longer reaction times and higher priming scores. Thought disorder was determined using the Positive and Negative Scales of PANSS that assess in the Positive Scale the presence of symptoms, as, for instance, hallucinations and conceptual disorganization, and in the Negative Scale the presence of symptoms, as, for instance, emotional withdrawal and difficulty in abstract thinking. The cognitive performance of people with SZ is also known to be modulated by the type of medication (typical *vs.* atypical drugs) and the illness duration ([24,60], but see [11,16]). Therefore, we also tested whether these clinical variables had any effects on the patients’ performance.

Consistent evidence has shown that the performance of SZ patients on phonemic and semantic fluency tests is almost always poor (for an overview, see [61]), with a larger deficit in semantic than in phonemic fluency tests. This has been thought to reflect a reduction of the size of the lexicon [62] and/or a dysfunction of the semantic system, since semantic fluency is more dependent on the integrity of semantic memory [61]. Therefore, we tested the association between the patients’ performance in verbal fluency tests and their response times and accuracy.

Finally, from a linguistic viewpoint, antonym word pairs differ in several respects. The first concerns whether there is only one canonical antonym to the word (canonical antonyms) or more than one (as in less clearly opposable antonyms as, for instance, white-dark, hot-iced; [32,38]). In this study, we only used canonical, one-to-one antonym pairs (e.g., dead-alive, black-white). Second, as we mentioned, “antonym” is a cover term for linguistically different members [31,32,63,64]. For instance, Jezek [64] claimed that Italian opposite words comprise: (1) “true” antonyms that are gradable adjectives lexicalizing two poles along a continuum that denotes a property or an event (e.g., easy/difficult); (2) binary oppositions (also called complementary or ancillary opposites [31]) that are mutually exclusive terms (e.g., dead/alive); and (3) converse terms (also called coordinated opposites [31]) in which there is a necessary relationship between terms (e.g., father/son). The linguistic study of Jezek [64] was not meant to analyze the processing differences among antonyms, binary oppositions and converse terms. Hence, to see whether these linguistic differences had any effects on the ability of patients and healthy controls to identify antonyms, we analyzed possible response times and accuracy differences among the three types of opposites. Third, the meaning conveyed by antonyms can vary along several dimensions, among which whether antonyms convey concrete or abstract meanings. This may be relevant, because not only healthy subjects usually take longer to process abstract rather than concrete concepts (for an overview, see [12,65,66]), but also because the processing disadvantage of abstract meanings is particularly evident in people with SZ [67,68,69]. Therefore, we tested whether concreteness had any effects on the ease with which patients and controls identified antonyms.

## 2. Methods

### 2.1. Participants

Participants included 39 chronic outpatients with paranoid SZ (DSM-V, [70] (14 female; mean age 31 years, age range 20–45, SD 6.2) and 39 healthy volunteers as control participants. Italian was the native language of all participants. The general inclusion criteria were at least 10 years of formal education and age between 18 and 45 years. Patients were recruited from the geographically defined catchment area of Modena and treated by the West Modena Mental Health Service and by a clinic reporting to the same mental health daycare district. Healthy control participants were volunteers recruited in the community through public advertisements. Controls were pairwise-matched to patients for age (±2), sex and education (±2) (see Table 1). Controls self-reported to have no history of alcohol or substance abuse, no major medical or neurological illness and no psychiatric illness in first degree relatives. To exclude any past or present psychiatric disorder, controls were administered the Brief Psychiatric Rating Scale (BPRS, [71]) by a senior psychiatrist. The diagnosis of paranoid SZ was based on the Positive and Negative Syndrome Scale (PANSS, [72]; score 46.69, range 34–68, SD 8.1), and it was confirmed by the clinical consensus of staff psychiatrists. The PANSS is a semi-structured interview designed to assess the presence and severity of positive, negative and general psychopathological symptoms by combining the patient’s scores in the Positive Syndrome Scale (7 items, e.g., hallucinations, conceptual disorganization), the Negative Syndrome Scale (7 items, e.g., emotional withdrawal, difficulty in abstract thinking) and the General Psychopathology Scale (16 items, e.g., anxiety, unusual thought content). The interview was administered to patients by senior psychiatrists blind to the cognitive data, and it was aimed at assessing the patients’ symptom status in the past week. Based on PANSS classification criteria, 35 patients had a mild form of SZ (PANSS total scores from 34 to 55) and four a moderate form (from 61 to 68). According to PANSS classification criteria, total scores up to 58 are indicative of a mild form of psychopathology and up to 75 of a moderate form. At the time of testing, all patients were responsive and clinically stabilized. None of them had comorbid psychiatric disorders, alcohol or substance abuse prior to the study, a history of traumatic head injury with loss of consciousness, epilepsy or other neurological diseases. Thirty-three of the 39 patients were prescribed second-generation antipsychotic medications (as defined by [73]); two were prescribed first-generation antipsychotics; and four were prescribed a combination of first- and second-generation antipsychotics. At the time of testing, patients had a mean IQ of 88 (range 58–126, SD 18) assessed with the Wechsler Adult Intelligence Scale-Revised (WAIS-R), a mean education of 12.6 years (range 10–14, SD 1.33) and a mean illness duration (from first diagnosis) of 8.97 years (range 1–29, SD 5.94) (see Table 1).

**Table 1:** Demographic characteristics of the study sample and clinical characteristics of the schizophrenic patients. WAIS-R, Wechsler Adult Intelligence Scale - Revised; BADA, *Batteria per l’analisi dei deficit afasici*; BPRS, Brief Psychiatric Rating Scale; PANSS, Positive and Negative Syndrome Scale.

Demographic/clinical criteria	Patients				Controls				*p*
	Mean	Min.	Max.	SD	Mean	Min.	Max.	SD	
Sex	M=25; F=14				M = 25; F = 14				
Age (years)	31.41	20	45	6.22	31.28	19	45	6.31	0.93
Education (years)	12.56	10	17	1.33	12.51	10	17	1.48	0.88
Drug	SG = 33; FG = 2; FSG = 4								
Years of illness	8.97	1	29	5.94					
WAIS-R (Verbal Scale)	91.05	62	118	15.41					
WAIS-R (Performance Scale)	86.31	58	121	19.42					
WAIS-R (total score)	87.82	58	126	18.31					
Vocabulary (WAIS-R)	8.23	3	15	3.24	10.77	7	17	2.38	0.0001
Phonemic fluency	28.51	15	54	8.25	37.28	23	58	7.68	0.0001
Semantic fluency	38.44	25	62	8.44	44.10	23	56	7.74	0.003
BADA (errors)	1.15	0	5	1.18	0.03	0	1	0.16	0.0001
Digit span (forward)	5.44	3.5	7.5	0.74	5.85	4.5	7.75	0.83	0.04
Digit span (backward)	3.75	1.69	6.42	1.07	4.28	1.47	6.47	0.97	0.05
Digit span (total score)	9.18	6.44	13.29	1,51	10.13	6.97	13.92	1.57	0.02
BPRS					2	2	2	0	
PANSS (Positive Scale)	11.64	7	19	3.12					
PANSS (Negative Scale)	11.21	7	26	4.02					
PANSS (General Psychopathology Scale)	23.84	18	34	3.43					
PANSS (Total Score)	46.69	34	68	8.13					

M = male; F = female; FG = first-generation antipsychotics; SG = second-generation antipsychotics; FSG = combination of first- and second-generation antipsychotics.

A set of neuropsychological tests was administered to patients and control participants to assess general cognitive functions and language (see Table 1). Specifically, patients were administered the WAIS-R, the syntactic competence sub-scale of the *Batteria per l’analisi dei deficit afasici* (BADA), originally designed for aphasic patients [74], to assess basic syntactic comprehension ability and the Phonemic and Semantic Fluency tests (Italian version; [75]). In SZ, verbal fluency impairments are thought to be indicative of a global intellectual deficit that affects different component processes and primarily semantic processes [61]. In the Phonemic Fluency Test, individuals produce as many words beginning with given letters (in Italian, F, P, L) as possible in a time interval (60 s for each letter). In the Semantic Fluency Test, individuals produce as many members of given stimulus categories (car brands, fruits and animals) as possible in a time interval (60 s for each category). For controls, the Digit Span and Vocabulary subtests of WAIS-R were used to estimate, respectively, verbal short-term memory and global verbal intelligence functions [76]. In the Vocabulary subtest of the WAIS, participants are asked to provide definitions for words of increasing rarity. This task is thought to measure crystallized verbal knowledge and is generally used to compare the clinical and control groups on previous exposure to and knowledge of word meanings. Controls were also administered the Phonemic and Semantic Fluency tests. The results of the neuropsychological tests showed that patients always had significantly poorer performances than controls (see Table 1). All subjects gave their informed consent for inclusion before they participated in the study. The study was conducted in accordance with the Declaration of Helsinki, and the protocol was approved by the Ethics Committee of Modena (Approval Code 180/07).

### 2.2. Materials

Sixty-five Italian antonym word pairs were selected from Italian dictionaries. The first word of each antonym pair was embedded in a definitional sentence fragment of the form, the opposite of word is... These sentence fragments were presented in a written form to 30 healthy participants (not involved in any subsequent phases of the experiment) who were asked to complete them. We selected the antonym pairs (Word 1 (W1)/W2) in which the antonym (W2) had the highest cloze probability value (*i.e.*, the proportion of participants that continued the definitional sentence fragment with that word; M = 0.98, SD = 0.6, range 0.69–1). This led to a stimulus set of 40 canonical antonym pairs, fourteen with an abstract meaning (e.g., right/wrong, easy/difficult, private/public) and 26 with a concrete meaning [77] (e.g., hot/cold, white/black, high/low) (see Appendix 1). Each W1 was paired with a semantically unrelated non-antonym target word (W3) that was never produced in cloze probability tests (see Appendix 1). In order to ensure that the effects of interest were not linked to specific word characteristics, antonym and non-antonym target words (W2s and W3s) were matched for length, frequency, grammatical class and age of acquisition (AoA) (see Table 2). To control for any further effects of the psycholinguistic characteristics of the stimuli, we correlated the response times to correct answers and the accuracy proportions with the mean number of letters, cloze probability, Age of Acquisition (AoA) and the written frequency of antonym and non-antonym target words for each group of participants. None of these correlations reached statistical significance. In contrast, correlations with the Google frequency of antonym word pairs revealed that the less frequent they were, the more time was necessary for controls to reject non-antonyms (*r* = −0.471, *p* = 0.002), and for patients to accept antonyms (*r* = −0.316, *p* = 0.047).

**Table 2:** Psycholinguistic characteristics of antonym and non-antonym target words.

Number of Letters			Age of Acquisition			Frequency (Ln)		
Opposite	Non-opposite	*p*	Opposite	Non-opposite	*p*	Opposite	Non-opposite	*p*
6.25 (1.69)	6.08 (1.49)	0.32	2.72 (0.97)	2.82 (1.07)	0.14	5.37 (1.37)	5.02 (1.60)	0.31

Two lists were created, each containing 40 sentences with the same format (the opposite of word is WORD, e.g., the opposite of black is WHITE/NICE). The target word was an antonym (W2, WHITE) in 20 sentences and a semantically unrelated, non-antonym word (W3, NICE) in the other 20 sentences, so that each W1 was presented only once in each list, either paired with an antonym (W2) or with a non-antonym (W3). The prime and target were antonyms in 50% of the trials and semantically unrelated in the other 50%. Participants were randomly assigned to one of the two lists. The order of presentation of the two lists was counterbalanced across participants.

### 2.3. Design and Procedure

The neuropsychological test administration (consisting of PANSS, WAIS-R, syntactic subscale of BADA and Verbal Fluency tests for patients; and of BPRS, Digit Span, Vocabulary and Verbal Fluency tests for controls) and the antonym experiment were carried out in different sessions (three for patients and two for controls) taking place a few days, one after the other. The order of test administration and experiment was randomized across participants.

each experimental trial began with a fixation cross (+) in the center of a computer screen. A spacebar press initiated the presentation of the prime word (W1) that was always embedded in a definitional sentence fragments as “the opposite of word is” (e.g., the opposite of black is). A second spacebar press initiated the presentation of the target word (WHITE/NICE), which remained on the screen until a response was given. The target was written in uppercase (Geneva bold, 14 font) and appeared in the center of the screen. Participants were instructed to press a “yes” button as quickly and accurately as possible if the target were correct or a “no” button if the target were not correct. The position of response buttons was counterbalanced across participants. The response times to target words were measured from the onset of the target word on the screen to the response. An experimenter sat behind the subject to ensure that s/he was pressing the spacebar to advance in the sentence presentation and the response buttons to respond (which always happened).

Each participant performed ten practice trials (not further used in the experiment), formed by five sentences ending with antonyms and five sentences with non-antonyms, followed by 40 experimental trials. Stimulus presentation and response collection were performed using a purpose-written E-Prime script (Psychology Software Tools).

### 2.4. Data Analysis

To examine potential confounding effects of verbal fluencies, vocabulary and digit span, analyses of covariance (ANCOVAs) were carried out on the mean response times to correct answers and on the mean proportions of correct responses. Group (patients *vs.* controls) was a between-subject factor and word type (antonym *vs.* non-antonym) a within-subject factor. Newman-Keuls *post hoc* tests were used to examine significant interactions (α = 0.05).

The effect of the definitional sentence fragment on the target word was operationalized in terms of a priming score (PRI) based on the response times to correct answers. The PRI of each participant was calculated as follows: (RT_unrelated targets_ – RT_related targets_)/RT_unrelated targets_) × 100 [54,56]. The individual PRIs of each patient and matched control were compared using an independent sample *t*-test.

Parametric bivariate correlations were conducted to examine the relationships between the patients’ mean response times, accuracy proportions and PRIs, the scores in the Positive and Negative Scale of PANSS and in the Verbal Scale of the WAIS-R. Non-parametric correlations (Spearman’s rho, r_s_) were conducted to examine the relationships between the patients’ response times, accuracy and PRIs and the type of medication, illness duration and number of items produced in the Semantic Fluency Test.

By-item ANOVAs (on response times and accuracy proportions) were used to test the concreteness effect with concreteness (abstract *vs.* concrete) as a between-item factor, group (patients *vs.* controls) and word type (antonym *vs.* non-antonym) as within-item factors.

Finally, the 40 antonym pairs of this study were divided into the three categories proposed by [64] for Italian (this led to 19 “true” antonyms, 10 binary oppositions and 11 converse terms). A by-item ANOVA was carried out with antonym type (antonym *vs.* binary *vs.* converse terms) as a between-item factor, group (patients *vs.* controls) and word type (antonym *vs.* non-antonym) as within-item factors.

## 3. Results and Discussion

**Figure 1:**
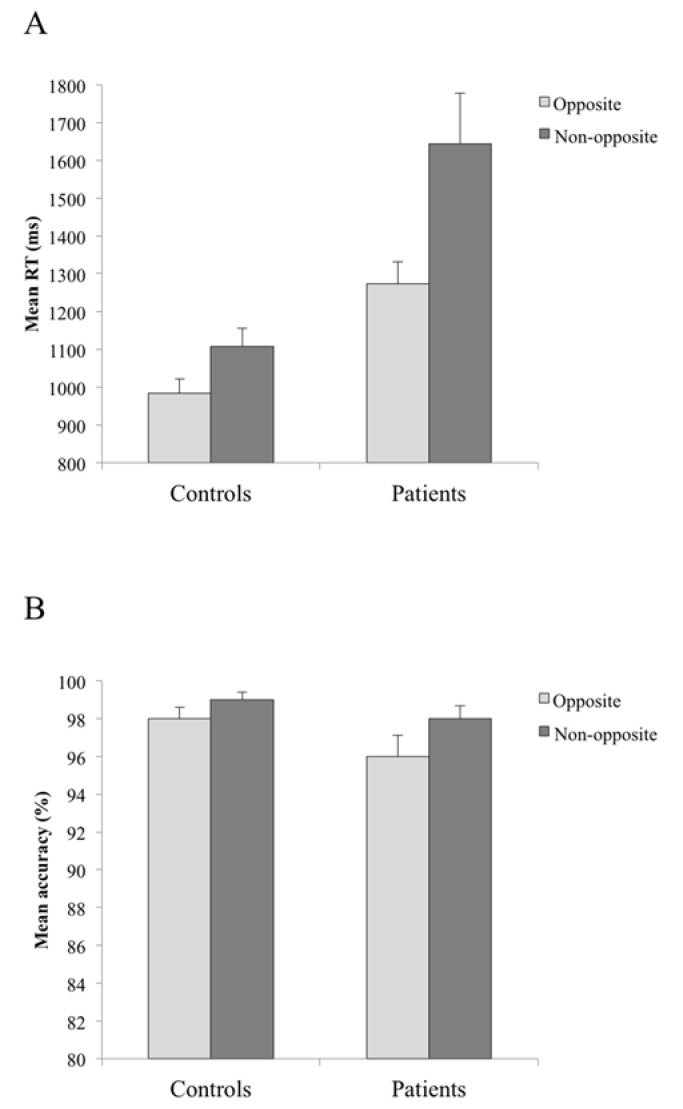
Mean reaction times (**A**) and mean percentage of correct responses (**B**) for controls and patients in opposite (bright gray bar) and non-opposite (dark gray bar) conditions. Bars represent standard errors of the mean.

The mean response times to correct responses and the accuracy proportions are shown in Figure 1.

The ANCOVA on response times to correct answers showed significant main effects of group (F(1,72) = 5.53, *p* < 0.02, ηp^2^= 0.07), with patients overall slower than controls, and of vocabulary (F(1,72) = 4.25, *p* < 0.04, ηp^2^= 0.06). The ANCOVA on accuracy showed a main effect of vocabulary (F(1,72) = 10.86, *p* < 0.002, ηp^2^= 0.13). Significant correlations between the vocabulary scores and the response times and accuracy proportions were obtained, but only for the patient group. Patients scoring higher in the vocabulary test also were faster at responding to antonyms and non-antonyms (*r* = −0.375, *p* = 0.019; *r* = −0.338, *p* = 0.035, respectively), and more accurate at rejecting non-antonyms (*r* = 0.331, *p* = 0.04). A significant correlation between the patients’ scores in the Verbal Scale of WAIS-R and response times showed that patients scoring higher on this scale also had faster response times to antonyms (*r* = −0.405, *p* = 0.011). No significant correlations emerged between the number of items produced by patients in the Semantic Fluency Test and their response times, PRIs and accuracy.

The analyses of the priming scores (PRIs) revealed an enhanced contextual priming in patients compared to controls (M =16.04%, SD = 19.4%; M = 9.6%, SD = 13.9%, respectively) (t(38) = −2.019, *p* = 0.05). No significant correlations emerged between PRIs and Positive Scale and Negative Scale scores.

The accuracy proportions negatively correlated with the Positive Scale scores in that patients scoring higher on this scale also had a lower accuracy on antonyms (*r* = − 0.363, *p* = 0.023). No significant correlations emerged between illness duration or type of medication and response times, PRIs and accuracy.

The by-item ANOVA on concreteness effects (we only comment on the effects significantly involving it) revealed a significant main effect of concreteness, with faster response times to concrete than abstract antonyms (M = 1166, SD = 161; M = 1294, SD = 287; respectively; F(1,38) = 6.505, MSe 91,031.02, *p* = 0.015), and a significant group by concreteness interaction (F(1,38) = 4.271, MSe 71,614.61, *p* = 0.046). Two separate ANOVAs were conducted on the response times of patients and controls. A significant main effect of concreteness emerged for both patients (F(1,38) = 6.921, MSe 77,707.764, *p* = 0.012) and controls (F(1,38) = 4.186, MSe 30,090.454, *p* = 0.048). Controls were 63 ms slower in correctly responding to abstract than to concrete antonyms controls, and patients were 172 ms slower. No significant effects of concreteness emerged on accuracy and PRIs.

Finally, the by-item ANOVAs considering the antonym type factor (we only comment on the effects significantly involving it) showed a significant main effect of this factor on response times (F(2,37) = 3.515, *p* = 0.04), with converse terms responded to faster than antonyms (t(49) = 2.353, *p* = 0.02). This might reflect the fact that both the semantic association between W1 and W2 and the written frequency of W2s were significantly higher in converse terms than in antonyms (t(28) = 3.571, *p* = 0.002; t(28) = 2.891, *p* = 0.007, respectively).

## 4. General Discussion

The general aim of the present investigation was to expand our knowledge of the cognitive processes underlying the recognition of antonyms and to evaluate whether these processes differed in SZ and in normal comprehension. Our results showed that antonym recognition was fast and accurate in healthy controls, in line with the literature. Important differences emerged between people with SZ and healthy controls. Specifically, the preceding definitional primes facilitated antonym recognition in both patients and healthy controls, but the amount of facilitation indeed differed. In fact, patients were helped more than controls by the previous definitional fragment, as shown by the larger reduction of response times to antonyms with respect to non-antonyms of patients (on average, patients were 25.4% faster in responding to antonyms than to non-antonyms, compared to 11.8% of controls). The definitional context preceding the target word had an exaggerated priming effect on patients, with close to twice the effect shown by controls (16.09% *vs.* 9.6%, respectively) (we return to this point below). Interestingly, this heightened semantic effect occurred under strategically controlled conditions (resulting from the definitional sentence fragment preceding the target and the self-paced nature of the task), rather than under the automatic condition typical of word priming for short SOAs [10]. This suggests a compromised ability of patients with SZ to engage in the controlled processing operations necessary to use in flexible ways semantic memory representations. The enhanced semantic priming of SZ patients was unrelated to the clinical state and to the thought disorder characterizing patients, as prior studies already have shown (e.g., [11,16,78]). In sum, patients seem to have encoded contextually relevant target words [21,22], but to a much higher degree than controls.

One might wonder whether this heightened priming effect can reflect the associative strength of the words forming the antonym pair. Unfortunately, word association was not tested in the norming phase. To overcome this limitation, we obtained ratings of semantic association, asking eleven healthy participants to produce the first word that came to mind for words of a list including our 40 W1s. On average, antonyms (W2s) were associated with W1s in 32.8% of the answers (SD 15.3; range 0–63). None of the non-antonym words (W3) was ever produced as an associate. No significant correlation emerged between PRIs and semantic association proportions. This makes it rather unlikely that the enhanced semantic priming effect of patients may reflect semantic association strength. Semantic association proportions instead significantly correlated with response times in that higher semantic association proportions led to faster responses to antonyms for both patients and controls (r = −0.342, *p* = 0.031; *r* = −435, *p* = 0.005, respectively).

Although patients made twice the number of errors of controls (127 *vs.* 62 errors), the group difference was not statistically significant (patients’ accuracy: 96.6%; controls: 98.5%). One possibility is that, indeed, processing polarity information is less demanding on executive resources than other types of semantic relationship [30]. However, we cannot exclude that the familiarity and strong coupling of the antonyms used in this study, together with the easy nature of the task, had minimized semantic processing demands, leading to a ceiling effect. An important within-patient difference on accuracy emerged in that patients with higher scores of positive thought disorder also had a diminished ability to recognize antonyms. This is consistent with the wealth of studies that have documented an association between thought disorder and semantic processing deficit (for an overview, see [2]). Accuracy instead increased in patients with high scores in the Vocabulary Subtest and in the Verbal Scale of WAIS-R (these patients also had faster response times). These results further confirm the observations that in SZ, high vocabulary scores are protective of semantic deterioration [15,28] and reflect premorbid intelligence [76]. As to the patients group, the association of vocabulary to the Verbal Scale scores is also consistent with the documented association of verbal intelligence to efficient language comprehension [79]. Surprisingly, we did not find a significant interaction between vocabulary and group, nor significant associations between the vocabulary scores and the response times and/or accuracy of healthy participants. One possibility is that the fast response times, compared to patients, and the high accuracy of healthy participants may have obscured the possibility of finding internal differences.

We wondered what the results would have looked like if people with SZ indeed were impaired in recognizing antonyms. Definitively, our results cannot be taken to mean that patients comprehended antonyms as controls, but simply at a slower pace. Rather, our results indicate that the state of residual SZ contributed to slower antonym recognition above and beyond the cognitive deficits that characterize SZ patients. Consistently, patients had an exaggerated reliance on stored lexico-semantic information and slowed down mechanisms of semantic search [20]. At the same time and unlike what was found in the few prior studies on antonyms in SZ, the relatively high level of accuracy of patients suggests that antonym recognition was not entirely lost in SZ. Models of semantic processing in SZ (for an overview, see [1,2,80]) have proposed that semantic deficit may arise from a variety of factors that includes disorganized or damaged semantic memory representations accompanied by impaired initial access to these representations [54,81,82,83]. Alternatively, semantic deficit may result from a compromised ability to engage in the controlled processing operations necessary to flexibly use semantic memory representations with preserved semantic storage and access to them [21,22,84]. Our results seem to be more compatible with the second than with the first model, since patients indeed comprehended the definitional sentence fragments and encoded the relevant targets, although they did it in an exaggerated way compared to controls, suggesting an abnormal ability to engage in the controlled processing operations required by the task and overreliance on stored semantic representations.

Antonyms are considered to be “conceptually identical in all respects but one, we perceive them as maximally similar, and, at the same time, due to the fact that they occupy radically different poles/parts, we perceive them as maximally different” [39] (p. 1052). All other things being equal, efficient and fast recognition of antonyms requires a preserved ability to appreciate the difference between maximally similar and maximally dissimilar meanings. The results of this study suggest that this ability depends on preserved executive resources, the integrity of the semantic processing system and the size of the lexicon. In fact, the healthy brain architecture subserving the comprehension of antonyms includes the regions of the left middle frontal gyrus that are generally engaged by retrieval mechanisms and semantic processing [47,85,86]. In addition, several studies reported that activation of these areas in SZ is predominantly abnormal [4,7,9].

There are some limitations to our study that must be addressed. Inclusion criteria resulted in a patient sample with low average levels of psychopathology (see Table 1). This may have limited the potential for detecting possible correlations with other clinical variables. In addition, patients were tested while they were clinically stabilized, hence limiting the conclusions that we can draw on the exact nature of the language processing perturbations in SZ. Finally, patients were on antipsychotic medication (although mostly on second-generation antipsychotic medication); hence, an effect of treatment could not be ruled out. Whatever the case, our results are still relevant insofar as they contributed to unveiling some of the mechanisms underlying the recognition of antonyms in both people with SZ and in healthy subjects.

## 5. Conclusions

Antonyms are considered to be “conceptually identical in all respects but one, we perceive them as maximally similar, and, at the same time, due to the fact that they occupy radically different poles/parts, we perceive them as maximally different” [39] (p. 1052). All other things being equal, efficient and fast recognition of antonyms requires a preserved ability to appreciate the difference between *maximally similar* and *maximally dissimilar* meanings [32]. The results of this study suggest that this ability depends on preserved executive resources, the integrity of the semantic processing system and the size of the lexicon. In fact, the healthy brain architecture subserving the comprehension of antonyms includes the regions of the left middle frontal gyrus that are generally engaged by retrieval mechanisms and semantic processing [47,85,86], but several studies reported that activation of these areas is predominantly abnormal in SZ [4,7,9].

## Appendix

**Appendix 1:** Complete list of opposite word pairs and unrelated targets (W1/W2/W3) used in the experiment.

W1	W2	W3
ottimista	pessimista	dilettante
inizio	fine	pelle
vivo	morto	forte
padre	madre	dolce
veloce	lento	sordo
giorno	notte	fuoco
falso	vero	solo
chiuso	aperto	presto
differente	uguale	lontano
maschio	femmina	contento
scuro	chiaro	perso
destra	sinistra	inverno
sotto	sopra	stanza
grande	piccolo	verde
brutto	bello	giusto
pesante	leggero	vicino
perdente	vincente	civile
vuoto	pieno	ricco
colpevole	innocente	commerciante
buono	cattivo	spento
privato	pubblico	critico
alto	basso	bravo
rumoroso	silenzioso	divertente
maggiore	minore	inutile
facile	difficile	fresco
lungo	corto	sporco
nuovo	vecchio	povero
luce	buio	mano
largo	stretto	viola
bianco	nero	caro
caldo	freddo	tenero
ottimo	pessimo	intatto
uomo	donna	carta
asciutto	bagnato	bugiardo
attivo	passivo	vorace
guerra	pace	gara
amore	odio	storto
fratello	sorella	favola
prima	dopo	come
giusto	sbagliato	colorato

## References

[1] Kuperberg G.R. (2010). Language in Schizophrenia. Part 1. An Introduction. Lang. Linguist. Compass.

[2] Kuperberg G.R. (2010). Language in Schizophrenia. Part 2. What psycholinguistics bring to the study of schizophrenia… and vice versa?. Lang. Linguist. Compass.

[3] Kiang M. (2010). Schizotypy and language: A review. J. Neurolinguist..

[4] Barch D.M., Ceaser A. (2012). Cognition in schizophrenia: Core psychological and neural mechanisms. Trends Cogn. Sci..

[5] Harvey P.D. (2010). Cognitive functioning and disability in schizophrenia. Curr. Dir. Psychol. Sci..

[6] Levy D.L., Coleman M.J., Sung H., Ji F., Matthysse S., Mendell N.R., Titone D. (2010). The genetic basis of thought disorder and language and communication disturbances in schizophrenia. J. Neurolinguist..

[7] Mitchell R.L., Crow T.J. (2005). Right hemisphere language functions and schizophrenia: The forgotten hemisphere?. Brain.

[8] Kuperberg G.R., Heckers S. (2000). Schizophrenia and cognitive function. Curr. Opin. Neurobiol..

[9] Kraguljac N.V., Srivastava A., Lahti A.C. (2013). Memory Deficits in Schizophrenia: A Selective Review of Functional Magnetic Resonance Imaging (fMRI) Studies. Behav. Sci..

[10] Minzenberg M.J., Ober B.A., Vinogradov S. (2002). Semantic priming in schizophrenia: A review and synthesis. J. Int. Neuropsychol. Soc..

[11] Pomarol-Clotet E., Oh T.M., Laws K.R., McKenna P.J. (2008). Semantic priming in schizophrenia: Systematic review and meta-analysis. Br. J. Psychiatry.

[12] Wang J., Conder J.A., Blitzer D.N., Shinkareva S.V. (2010). Neural representation of abstract and concrete concepts: A meta-analysis of neuroimaging studies. Hum. Brain Mapp..

[13] Mathalon D.H., Roach B.J., Ford M. (2010). Automatic semantic priming abnormalities in schizophrenia. Int. J. Psychophysiol..

[14] Kiang M., Christensen B.K., Kutas M., Zipursky R.B. (2012). Electrophysiological evidence for primary semantic memory functional organization deficits in schizophrenia. Psychiatry Res..

[15] Brébion G., Stephan-Otto C., Huerta-Ramos E., Usall J., Ochoa S., Roca M., Abellan-Vega H., Haro J.M. (2013). Abnormal functioning of the semantic network in schizophrenia patients with thought disorganization. An exemplar production task. Psychiatry Res..

[16] Ditman T., Goff D., Kuperberg G. (2011). Slow and steady: Sustained effects of lexico-semantic associations can mediate referential impairments in schizophrenia. Cogn. Affect. Behav. Neurosci..

[17] Cohen J.D., Barch D.M., Carter C., Servan-Schreiber D. (1999). Context-processing deficits in schizophrenia: Converging evidence from three theoretically motivated cognitive tasks. J. Abnorm. Psychol..

[18] Kuperberg G.R., McGuire P.K., David A.S. (1998). Reduced sensitivity to linguistic context in schizophrenic thought disorder: Evidence from on-line monitoring for words in linguistically anomalous sentences. J. Abnorm. Psychol..

[19] McCarley R.W., Niznikiewicz M.A., Salisbury D.F., Nestor P.G., O’Donnell B.F., Hirayasu Y., Grunze H., Greene R.W., Shenton M.E. (1999). Cognitive dysfunction in schizophrenia: Unifying basic research and clinical aspects. Eur. Arch. Psychiatry Clin. Neurosci..

[20] Niznikiewicz M.A., Friedman M., Shenton M.E., Voglmaier M., Nestor P.G., Frumin M., Seidman L., Sutton J., McCarley R.W. (2004). Processing sentence context in women with schizotypal personality disorder: An ERP study. Psychophysiology.

[21] Titone D., Levy D.L., Holzman P.S. (2000). Contextual insensitivity in schizophrenic language processing: Evidence from lexical ambiguity. J. Abnorm. Psychol..

[22] Titone D., Holzman P.S., Levy D.L. (2002). Idiom processing in schizophrenia: Literal implausibility saves the day for idiom priming. J. Abnorm. Psychol..

[23] Cohen J.D., Servan-Schreiber D. (1992). Context, cortex, and dopamine: A connectionist approach to behavior and biology in schizophrenia. Psychol. Rev..

[24] Barch D.M., Cohen J.D., Servan-Schreiber D., Steingard S., Steinhauer S.S., van Kammen D.P. (1996). Semantic priming in schizophrenia: An examination of spreading activation using word pronunciation and multiple SOAs. J. Abnorm. Psychol..

[25] Salisbury D.F. (2004). Semantic memory and verbal working memory correlates of N400 to subordinate homographs. Brain Cogn..

[26] Ditman T., Kuperberg G.R. (2007). The time course of building discourse coherence in schizophrenia: An ERP investigation. Psychophysiology.

[27] Condray R., Steinhauer S.R., van Kammen D.P., Kasparek A. (2002). The language system in schizophrenia: Effects of capacity and linguistic structure. Schizophr. Bull..

[28] Brébion G., Bressan R.A., Ohlsen R.I., Pilowsky L.S., David A.S. (2010). Production of atypical category exemplars in patients with schizophrenia. J. Int. Neuropsychol. Soc..

[29] Ditman T., Kuperberg G. (2010). Building coherence: A framework for exploring the breakdown of links across clause boundaries in schizophrenia. J. Neurolinguist..

[30] Crutch S.J., Williams P., Ridgway G.R., Borgenicht L. (2012). The role of polarity in antonym and synonym conceptual knowledge: Evidence from stroke aphasia and multidimensional ratings of abstract words. Neuropsychologia.

[31] Jones S. (2002). Antonymy: A Corpus-Based Perspective.

[32] Paradis C., Willners C. (2011). Antonymy: From convention to meaning-making. Rev. Cogn. Linguist..

[33] Bianchi I., Savardi U., Kubovy M. (2011). Dimensions and their poles: A metric and topological theory of opposites. Lang. Cogn. Process..

[34] Fellbaum C., Fellbaum C. (1998). WordNet: An Electronic Lexical Database.

[35] Willners C. (2001). Antonyms in Context. A Corpus-Based Semantic Analysis of Swedish Descriptive Adjective. Travaux de l’Institut de Linguistique de Lund 40.

[36] Murphy M.L. (2003). Semantic Relations and the Lexicon: Antonymy, Synonymy and Other Paradigms.

[37] Van de Weijr J., Paradis C., Willners C., Lindgren M. (2014). Antonym canonicity: Temporal and contextual manipulations. Brain Lang..

[38] Paradis C., Willners C., Jones S. (2009). Good and bad opposites: Using textual and psycholinguistic techniques to measure antonym canonicity. Mental Lex..

[39] Gross D., Fischer U., Miller G.A. (1989). The organization of adjectival meanings. J. Memory Lang..

[40] Blumberg S., Giller D.W. (1965). Some verbal aspects of primary-process thought: A partial replication. J. Personal. Soc. Psychol..

[41] Burstein A.G. (1961). Some verbal aspects of primary process thought in schizophrenia. J. Abnorm. Soc. Psychol..

[42] Bentin S. (1987). Event-related potentials, semantic processes, and expectancy factors in word recognition. Brain Lang..

[43] Kretzschmar F., Bornkessel-Schlesewsky I., Schlesewsky M. (2009). Parafoveal and foveal N400s dissociate spreading activation from contextual fit. NeuroReport.

[44] Roehm D., Bornkessel-Schlesewsky I., Rösler F., Schlesewsky M. (2007). To predict or not to predict: Influences of task and strategy on the processing of semantic relations. J. Cogn. Neurosci..

[45] Hutchison K.A. (2003). Is semantic priming due to association strength or feature overlap? A microanalytic review. Psychon. Bull. Rev..

[46] Arnaud P.J.L. (1999). Target-error resemblance in French word substitution speech errors and the mental lexicon. Appl. Psycholinguist..

[47] Jeon H.A., Lee K.M., Kim Y.B., Cho Z.H. (2009). Neural substrates of semantic relationships: Common and distinct left-frontal activities for generation of synonyms *vs.* antonyms. NeuroImage.

[48] Kutas M., Iragui V. (1998). The N400 in a semantic categorization task across six decades. Electroencephalogr. Clin. Neurophysiol..

[49] Just M.A., Carpenter P.A. (1980). A theory of reading: From eye fixations to comprehension. Psychol. Rev..

[50] Kuperberg G.R., Kreher D.A., Goff D., McGuire P.K., David A.S. (2006). Building up linguistic context in schizophrenia: Evidence from self-paced reading. Neuropsychology.

[51] Butler P.D., DeSanti L.A., Maddox J., Harkavy-Friedman J.M., Amador X.F., Goetz R.R., Javitt D.C., Gorman J.M. (2002). Visual backward-masking deficits in schizophrenia: Relationship to visual pathway function and symptomatology. Schizophr. Res..

[52] Quelen F., Grainger J., Raymondet P. (2005). An investigation of semantic priming in schizophrenia using a new priming paradigm. Schizophr. Res..

[53] Vinogradov S., Poole J.H., Willis-Shore J., Ober B.A., Shenaut G.K. (1998). Slower and more variable reaction times in schizophrenia: What do they signify?. Schizophr. Res..

[54] Spitzer M., Braun U., Hermle L., Maier S. (1993). Associative semantic network dysfunction in thought-disordered schizophrenic patients: Direct evidence from indirect semantic priming. Biol. Psychiatry.

[55] Spitzer M., Weisker I., Winter M., Maier S., Hermle L., Maher B.A. (1994). Semantic and phonological priming in schizophrenia. J. Abnorm. Psychol..

[56] Kiefer M., Martens U., Weisbrod M., Hermle L., Spitzer M. (2009). Increased unconscious semantic activation in schizophrenia patients with formal thought disorder. Schizophr. Res..

[57] Safadi Z., Lichtenstein-Vidne L., Dobrusin M., Henik A. (2013). Investigating thought disorder in schizophrenia: Evidence for pathological activation. PLoS One.

[58] Salisbury D.F. (2008). Semantic activation and verbal working memory maintenance in schizophrenic thought disorder: Insights from electrophysiology and lexical ambiguity. Clin. EEG Neurosci..

[59] Sitnikova T., Perrone C., Goff D., Kuperberg G.R. (2010). Neurocognitive mechanisms of conceptual processing in healthy adults and patients with schizophrenia. Int. J. Psychophysiol..

[60] Maher B.A., Manschreck T.C., Redmond D., Beaudette S. (1996). Length of illness and the gradient from positive to negative semantic priming in schizophrenic patients. Schizophr. Res..

[61] Henry J.D., Crawford J.R. (2005). A meta-analytic review of verbal fluency deficits in schizophrenia relative to other neurocognitive deficits. Cogn. Neuropsychiatry.

[62] Chen R.Y.L., Chen E.Y.H., Chan C.K.Y., Lam L.C.W., Lieh-Mak F. (2000). Verbal fluency in schizophrenia: Reduction in semantic store. Aust. New Zealand J. Psychiatry.

[63] Cruse D.A. (1976). Three classes of antonym in English. Lingua.

[64] Jezek E. (2005). Lessico. Classi di Parole, Strutture e Combinazioni.

[65] Duñabeita J.A., Avilés A., Afonso O., Scheepers C., Carreiras M. (2009). Qualitative differences in the representation of abstract *versus* concrete words: Evidence from the visual-world paradigm. Cognition.

[66] Kousta S.T., Vigliocco G., Vinson D.P., Andrews M., del Campo E. (2011). The representation of abstract words: Why emotion matters. J. Exp. Psychol..

[67] Gorham D.R. (1961). Verbal abstraction in psychiatric illness: Assay of impairment utilizing proverbs. Br. J. Psychiatry.

[68] Schettino A., Lauro L.R., Crippa F., Anselmetti S., Cavallaro R., Papagno C. (2010). The comprehension of idiomatic expressions in schizophrenic patients. Neuropsychologia.

[69] Pesciarelli F., Gamberoni T., Ferlazzo F., lo Russo L., Pedrazzi F., Melati E., Cacciari C. (2014). Is the comprehension of idiomatic sentences indeed impaired in paranoid schizophrenia? A window into semantic processing deficits. Front. Hum. Neurosc..

[70] American Psychiatric Association (2013). Diagnostic and Statistical Manual of Mental Disorders.

[71] Ventura J., Lukoff D., Nuechterlein K.H., Liberman R.P., Green M.E., Shaner A. (1993). Brief Psychiatric Rating Scale (BPRS), Expanded Version (4.0): Scales, anchor points, and administration manual. Int. J. Methods Psychiatric Res..

[72] Kay S.R., Fisz-Bein A., Opler L.A. (1987). The Positive and Negative Syndrome Scale (PANSS) for schizophrenia. Schizophr. Bull..

[73] Lohr J.B., Braff D.L. (2003). The value of referring to recently introduced antipsychotics as “second generation”. Am. J. Psychiatry.

[74] Miceli G., Laudanna A., Burani C., Capasso R. (1994). Batteria per l’Analisi dei Deficit Afasici (B.A.D.A.).

[75] Novelli G., Papagno C., Capitani E., Laiacona N., Vallar G., Cappa S.F. (1986). Tre test clinici di ricerca e produzione lessicale. Taratura su soggetti normali. Arch. Psicol. Neurol. Psichiatr..

[76] Lezak M.D., Howieson D.B., Loring D.W. (2004). Neuropsychological Assessment.

[77] Della Rosa P., Catricalà E., Vigliocco G., Cappa S. (2010). Beyond the abstract–concrete dichotomy: Mode of acquisition, concreteness, imageability, familiarity, age of acquisition, context availability, and abstractness norms for a set of 417 Italian words. Behav. Res. Methods.

[78] Lecardeur L., Giard B., Mickael Laisney M., Brazo P., Delamillieure P., Eustache F., Dollfus S. (2007). Semantic hyperpriming in schizophrenic patients: Increased facilitation or impaired inhibition in semantic association processing?. Schizophr. Res..

[79] Hunt E., Anderson R.C., Spiro R.J., Montague W.E. (1977). We know who knows, but why?. Schooling and the Acquisition of Knowledge.

[80] Titone D., Libben M., Niman M., Ranbom L., Levy D.L. (2007). Conceptual combination in schizophrenia: Contrasting property and relational interpretations. J. Neurolinguist..

[81] Aloia M.S., Gourovitch M.L., Missar D., Pickar D., Weinberger D.R., Goldberg T.E. (1998). Cognitive substrates of thought disorder: Specifying a candidate cognitive mechanism. Am. J. Psychiatry.

[82] Chen E.Y., Wilkins A.J., McKenna P.J. (1994). Semantic memory is both impaired and anomalous in schizophrenia. Psychol. Med..

[83] Elvevag B., Weickert T., Wechsler M., Coppola R., Weinberger D.R., Goldberg T.E. (2002). An investigation of the integrity of semantic boundaries in schizophrenia. Schizophr. Res..

[84] Sitnikova T., Salisbury D.F., Kuperberg G., Holcomb P.I. (2002). Electrophysiological insights into language processing in schizophrenia. Psychophysiology.

[85] Poldrack R.A., Wagner A.D., Prull M.W., Desmond J.E., Glover G.H., Gabrieli J.D.E. (1999). Functional specialization for semantic and phonological processing in the left inferior frontal cortex. NeuroImage.

[86] Price C.J., Moore C.J., Humphreys G.W., Wise R.S.J. (1997). Segregating semantic from phonological processes during reading. J. Cogn. Neurosci..

